# Alkaloids from *Aconitum carmichaelii* Alleviates DSS-Induced Ulcerative Colitis in Mice *via* MAPK/NF-*κ*B/STAT3 Signaling Inhibition

**DOI:** 10.1155/2022/6257778

**Published:** 2022-05-31

**Authors:** Chuanqi Huang, Junli Dong, Lu Cheng, Haoran Ma, Fuqian Wang, Yan Feng, Shu Zhang, Yuling Li, Dan Zhang, Xiaoqi Jin, Xin Xiong, Jie Jiang, Bin Wu, Hongfeng Xu, Geng Zhang

**Affiliations:** ^1^Department of Pharmacy, Wuhan No. 1 Hospital (Wuhan Hospital of Traditional and Western Medicine), Wuhan 430022, China; ^2^Department of Pathology, Wuhan No. 1 Hospital (Wuhan Hospital of Traditional and Western Medicine), Wuhan 430022, China; ^3^Department of Gastroenterology, Wuhan No. 1 Hospital (Wuhan Hospital of Traditional and Western Medicine), Wuhan 430022, China; ^4^College of Materia Medica, Shenyang Pharmaceutical University, Shenyang 110016, China; ^5^Department of Transfusion Medicine, Wuhan No. 1 Hospital (Wuhan Hospital of Traditional and Western Medicine), Wuhan 430022, China

## Abstract

Fuzi (*Aconitum carmichaelii* Debx) has been traditionally used for the treatment of ulcerative colitis (UC) in China for thousands of years. The total alkaloids of *A. carmichaelii* (AAC) have been considered as the main medicinal components of fuzi, whereas its underlying anti-UC mechanisms remain elusive. In the present study, the dextran sulfate sodium (DSS)-induced UC mice model, which was consistent with the symptoms and pathological features of human UC, was established to comprehensively evaluate the anti-UC effects of AAC. The results indicated that AAC effectively improved the weight loss, disease activity index (DAI), spleen hyperplasia, and colon shortening, and thus alleviated the symptoms of UC mice. Meanwhile, AAC not only inhibited the MPO enzyme and the abnormal secretion of inflammatory cytokines (TNF-*α*, IL-1*β*, IL-6, IFN-*γ*, and IL-17A) and suppressed the overexpression of inflammatory mediators (TNF-*α*, IL-1*β*, and IL-6) of mRNA but also reduced the phosphorylation of p38 MAPK, ERK, and JNK, and the protein expressions of NF-*κ*B, I*κ*B-*α*, STAT3, and JAK2 in the colon tissue. Furthermore, the LC-MS/MS quantitative determination suggested that the three low toxic monoester alkaloids were higher in both contents and proportion than that of the three high toxic diester alkaloids. Additionally, molecular docking was hired to investigate the interactions between alkaloid-receptor complexes, and it suggested the three monoester alkaloids exhibited higher binding affinities with the key target proteins of MAPK, NF-*κ*B, and STAT3. Our finding showcased the noteworthy anti-UC effects of AAC based on the MAPK/NF-*κ*B/STAT3 signaling pathway, which would provide practical and edge-cutting background information for the development and utilization of *A. carmichaelii* as a potential natural anti-UC remedy.

## 1. Introduction

Inflammatory bowel disease (IBD), a type of intestinal inflammatory disease with complex etiology and pathogenesis, mainly includes Crohn's disease (CD) and ulcerative colitis (UC) [1]. In recent decades, the global incidence of IBD has been increasing year by year. It is estimated that the incidence in the United States and Europe has reached 1 million and 2.5 million [2] in which the incidences of UC and CD in Europe are as high as 24/100,000 and 11.5/100,000 [3], respectively. In addition, the total cases of IBD in China from 2005 to 2014 were about 350,000 according to the Chinese Center for Disease Control and Prevention (CCDCP), and it is expected to reach 1.5 million in 2025 [4]. Particularly, China has the highest incidence of IBD of 3.44/100,000 individuals in Asia [5].

UC is a chronic nonspecific intestinal inflammatory disease, mainly affecting the ileum and colon. Its common clinical symptoms are persistent diarrhea, abdominal cramps, and rectal bleeding, accompanied by the loss of appetite and weight loss [6]. Currently, UC is mainly considered to be an autoimmune-related disease [7]. Meanwhile, a lot of reports indicate that the interaction of intestinal microbes, intestinal epithelial cells, and immune system are also involved in the pathogenesis of UC [8–11]. Due to the recurrent attacks, UC has posed a great impact on patients' quality of life and become one of the major intestinal diseases harmful to public health in recent years.

Generally, the onset of UC is closely related to the activation of MAPK, NF-*κ*B, and STAT3 signaling pathways. For example, NF-*κ*B is a downstream signal molecule of p38 MAPK, and its signal transduction is involved in the regulation of a large number of genes during the inflammatory response of human UC. The activation of p38 MAPK might trigger the NF-*κ*B signaling pathway, prompting NF-*κ*B to enter the nucleus to regulate downstream target inflammatory genes, such as IL-6, IL-10, and TNF-*α*. These cytokines induce the production of reactive oxygen species (ROS) and cause a strong and long-term inflammatory response [12]. Furthermore, studies have shown that cytokines and growth factors bind to tyrosine kinase (TK)-related receptors to activate JAK kinases and STAT transcription factor family; that is, through Janus kinase-signal transduction and transcription activation, the Janus kinase-signal transducer and activator of transcription (JAK-STAT) pathway play a biological role and regulate basic immune processes, thus exhibiting a key role in both intestinal homeostasis and UC [13]. For instance, mice with intestinal epithelial cells (IECs)-specific STAT3 gene defects are more likely to be induced by chemical agents to form experimental colitis, leading to epithelial erosion and mucosal inflammation, thus suggesting that JAK-STAT plays an important role in the incidence of UC [14, 15].

Currently, a number of drugs have been used clinically for the treatment of UC, including sulfasalazine (SASP), 5-aminosalicylic acid (5-ASA), glucocorticoids, and anti-TNF-*α* monoclonal antibodies. Although they have displayed palliative effects on UC, a series of factors such as the low effective rate of SASP and 5-ASA, glucocorticoid easily causing adverse reactions such as glucose disorder, and the high price of monoclonal antibody limit their wide clinical application [16]. Therefore, finding new and more effective remedies is of importance to improve the prognosis of UC patients. To this point, the traditional Chinese medicine (TCM) has indicated good effects for the treatment of UC, including *Indigo naturalis* [17], *Chrysanthemum morifolium* [18], *Morinda officinalis* [19], Gegen Qinlian decoction [20], and Lizhong decoction [21], with advantages of multitarget, overall regulation, limited side effects, and stable efficacy.

In our previous study, fuzi (*Aconitum carmichaelii* Debx) decoction exerted excellent overall or partial inhibitory and activation effects on the MAPK, NF-*κ*B, and STAT3 signaling pathways, thus posing obvious anti-UC activity [22]. However, there is still no clear evidence about which components (groups) play this activity in fuzi decoction. Based on the Chinese Pharmacopoeia and our previous study, the total alkaloids of *A. carmichaelii* (AAC) are likely to be the highly active ingredients in fuzi with anti-UC. Hence, as the ongoing study of fuzi on the treatment of UC, the present work continues to comprehensively investigate the palliative and ameliorating effects of AAC on UC mice model and analyze the underlying mechanisms based on the MAPK/NF-*κ*B/STAT3 signaling pathways, so as to provide practical guidance for the clinical and daily usages of this valuable alkaloid-rich natural resources, and the development of new natural anti-UC pharmaceuticals in near future.

## 2. Materials and Methods

### 2.1. Animals

Specific pathogen-free (SPF) male C57BL/6 mice (6 to 8 weeks old, weighting 20 to 22 *g*) were provided by Hubei Provincial Laboratory Animal Research Center (Wuhan, Hubei, China). The mice were group-housed under SPF condition in a 12-h light/dark cycle with food water *ad libitum*. Formal experiments were conducted after 7 days of adaptive feeding, and all experimental protocols and procedures were followed with the approval by European Community Guidelines, the regulations of the National Institute of Health of USA, and the Animal Care and Welfare Committee of Wuhan No. 1 Hospital (approval number: 2017022).

### 2.2. Preparation for Alkaloids of *Aconitum carmichaelii* (AAC)

High-grade aconiti lateralis radix praeparata (seminal root of *A. carmichaelii* Debx.) was purchased from Tianji pharmaceutical Ltd. (Wuhan, Hubei, China), and voucher specimens (No. 20171201) were deposited in the Chinese medicine herbarium of Wuhan No. 1 Hospital. The aconiti lateralis radix praeparata was grounded as coarse powder (24 mesh), soaked in hydrogen chloride solution (pH 4) at the ratio (weight/volume) of 1 : 10 for 6 h, and extracted by boiling for 2 h. The extracts were collected, filtered, and adjusted to pH 10 with 10 M of NaOH. After fully blending the solution, equal volume ethyl acetate was added to extracts and the ethyl acetate layer was collected. After ethyl acetate was volatilized in a water bath at 50°C, 0.5 mL ethanol was added to dissolve alkaloids, and an appropriate amount of distilled water was added according to the crude drug dose (3 g/kg for AAC-L group, 6 g/kg for AAC-M group, and 12 g/kg for AAC-H group in body weight) [22] to prepare the corresponding concentrations of AAC solutions, which were frozen at −80°C before gavage.

### 2.3. LC-MS/MS Analysis of AAC

In the present study, the 6 referential alkaloids (aconitine, hypaconitine, mesaconitine, benzoylaconine, benzoylhypaconine, and benzoylmesaconine) of *A. carmichaelii* recorded in Chinese Pharmacopoeia in AAC-L were determined by LC-MS/MS. The analyses were performed using an LC-20AD system (Shimadzu Corp., Kyoto, Japan) equipped with a VP-ODS chromatographic column (4.6 mm × 250 mm, 5 *μ*m, GL Science, Torrance, CA, USA) and connected with an API 4000 quadrupole mass spectrometer (Applied Biosystems, Waltham, MA, USA). The chromatographic and mass spectrometry conditions were consistent with those of previous study [22].

### 2.4. Induction of Ulcerative Colitis and Treatment

**Figure 1:**
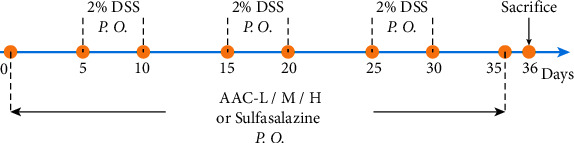
Model induction and treatment of ulcerative colitis in mice.

A total of 48 mice were randomly divided into 6 groups, namely, normal control (NC), dextran sulfate sodium (DSS), sulfasalazine (SASP), low-dose of alkaloids from *A. carmichaelii* (AAC-L), medium-dose of alkaloids from *A. carmichaelii* (AAC-M), and high-dose of alkaloids from *A. carmichaelii* (AAC-H) groups. According to the drug administration schedule (Figure 1), only distilled water was fed in NC group throughout the experiment, while in other groups, distilled water and 2% DSS aqueous solution were fed alternately for 5 days and continued for 35 days to induce the UC model. In addition, mice in each group were given different therapeutic drugs or placebo: NC and DSS groups were gavage with 20 mL/kg normal saline, SASP group was gavage with 200 mg/kg sulfasalazine, and AAC-L/M/H groups were gavage with 3, 6, and 12 g/kg crude drug dose of AAC, respectively.

### 2.5. Evaluation of Disease Activity Index (DAI)

DAI is an important method to evaluate the clinical symptoms of UC in experimental animals, and this index is calculated according to the following formula [23]: DAI = (weight loss score + stool character score + bloody score)/3. On day 36 of the experiment, blood was collected from the hearts of the mice under phenobarbital anesthesia, followed by colon and proximal rectum collection and measurement of colon length and weighing of the spleen. Each segment of colon tissue was divided into several parts and frozen at −80°C for later use.

### 2.6. Determination of MPO and Inflammatory Cytokines

IL-1*β*, IL-6, IL-10, IL-17A, TNF-*α*, IFN-*γ*, and MPO, in the colon tissue homogenate, were measured in by enzyme-linked immunosorbent assay (ELISA) according to the manufacturer's instructions (eBioscience, San Diego, CA, USA). Catalog numbers were 88-7013-22, 88-7064-22, 88-7105-22, BMS6001, 88-7324-22, 88-8314-22, and EMMPO, respectively. In brief, total protein of each colonic tissue was obtained by lysates in an ice bath, and the supernatant was collected and centrifuged at 1,2000 × *g* at 4°C for 10 min. Then, the protein content was measured with BCA protein kit (Jiancheng Bioengineering Institute, Jiangsu, China), and the above cytokines and MPO contents were measured by taking the same amount of colonic protein from each mouse.

### 2.7. Quantitative Real-Time PCR

After homogenizing colon tissue in ice bath, total RNA was extracted by TRIzol® (Invitrogen, Waltham, MA, USA), and Reverse Transcriptase M-MLV kit (TAKARA Bio., Shiga, Kansai, Japan) was used to synthesize cDNA. The relative contents of TNF-*α*, IL-1*β*, and IL-6 mRNA of mice in each group were calculated by quantitative real-time PCR under SYBR® Premix EX Taq™ (TAKARA Bio., Shiga, Kansai, Japan) kit according to 2^−△△Ct^. The PCR amplification conditions were as follows: 5 min at 95°C, followed by 42 cycles at 95°C for 12 s and 60°C for 31 s. Oligonucleotide sequences of primers (Sangon Biotech, Shanghai, China) for TNF-*α*, IL-1*β*, IL-6, and *β*-actin are listed in Table 1.

### 2.8. Western Blotting

The colon tissues were pestled into powder in liquid nitrogen, and the total proteins were extracted with ice-cold RIPA protein extraction kit (Thermo Fisher scientific, MA, USA). After centrifugation at 10,000 × *g* at 4°C for 10 min, the supernatants were collected to obtain the protein extracts and the concentration of each sample was determined with BCA assay kit (Jiancheng Bioengineering Institute, Jiangsu, China). The proteins were separated by sodium dodecyl sulfonate-polyacrylamide gel electrophoresis (SDS-PAGE) and electrotransferred to the polyvinylidene fluoride (PVDF) membrane (0.45 mm, Millipore, MA, USA). The proteins were incubated at 4°C overnight with STAT3 (STAT3, JAK1, JAK2, and JAK3), MAPK (p-ERK, ERK, p-JNK, JNK, p38 MAPK, and p-p38 MAPK), or NF-*κ*B (NF-*κ*B p65 and I*κ*B-*α*) primary antibodies (1 : 1000, Cell Signaling Technology, KY, USA) and then incubated with corresponding secondary antibody (1 : 4000) at room temperature for 1 h. The membranes were washed for 3 times with Tris-buffer [contain 0.1% (v/v) Tween^®^ 20] and incubated with enhanced chemiluminescence (ECL) reagent (Millipore, MA, USA). The protein bands were captured with X-ray films and quantified by Imaging System (Bio-Rad, Hercules, CA) using glyceraldehyde 3-phosphate dehydrogenase (GAPDH) as an internal reference control.

### 2.9. Histopathological Examination

The colon tissues were fixed overnight in 10% neutral formalin buffer solution at 4°C, thoroughly rinsed with tap water for 3 times, and then dehydrated with gradient concentrations of ethanol. The samples were soaked with xylene, embedded with paraffin, cut into 4-*μ*m slices, stained with hematoxylin and eosin, and observed under a microscope (BX53, Olympus, Shinjuku, Tokyo, Japan) to analyze the pathological conditions of samples in each group. Histological results were calculated according to a combined score criteria of inflammatory cell infiltration and tissue damage [21].

### 2.10. Molecular Docking

The molecular docking simulation between MAPK (PDB : 1A9U), NF-*κ*B (PDB : 1NFI), and STAT3 (PDB : 1BG1) with 6 alkaloids was implemented with the computational program AutoDock Vina (v 1.1.2, National Biomedical Computation Resource, San Diego, CA, USA). Firstly, the 3D structures of MAPK, NF-*κ*B, and STAT3 were downloaded from the RCSB protein databank (http://www.rcsb.org). The 3D structures of 6 alkaloids with the lowest energy were established by using ChemBio3D Ultra (v 14.0, Cambridge Soft Corp., Cambridge, MA, USA) and were transferred to pdb format. After that, the receptors and ligands were prepared by removing water molecules, adding hydrogen atoms, calculating the charge, and confirming the protonation state with Autodock Tools (v 4.2) and then converted to the readable pdbqt format. Subsequently, a sphere with a radius of 10 Å was centered on the active site of MAPK/NF-*κ*B/STAT3, and the AutoGrid processing was performed to set the grid box with the dimension size of 60 × 60 × 60, and with the centroid coordinates: x_1.607, y_8.382, z_31.651 for MAPK; x_2.585, y_55.137, z_-28.829 for NF-*κ*B; x_115.024, y_87.361, and z_-56.162 for STAT3. Finally, the AutoDock calculations were conducted through 2.5 × 10^6^ energy assessments, coupled with 50 independent runs of genetic algorithms in a flexible way. The parameters above are default parameters unless specified. PyMOL Molecular Visualization 2.2 (DeLano Scientific LLC, San Carlos, CA, USA) and AutoDock Tools were applied to observe and obtain all visual 3D geometry and docking results.

### 2.11. Statistics

Statistical analysis was performed by one-way analysis of variance (ANOVA), followed by Tukey's multiple comparison tests for calculating intergroup differences with SPSS 20.0 (SPSS Inc., Chicago, IL, USA). The difference between the means was considered statistically significant at *p* < 0.05.

## 3. Results

### 3.1. Effects of AAC on Clinical Indices

**Figure 2:**
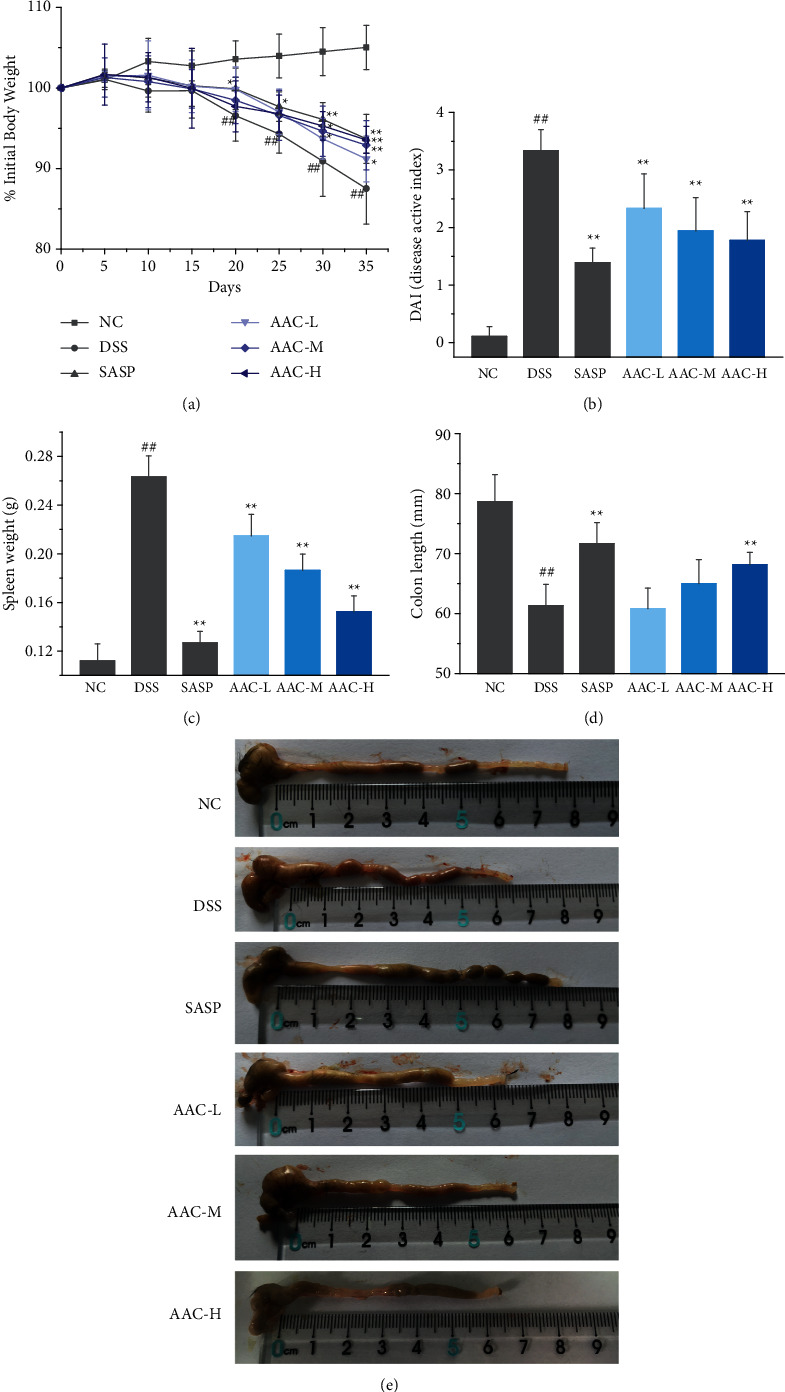
Effects of AAC on clinical indices. (a) Percentage changes in body weight compared to initial weight; (b) disease activity index (DAI); (c) spleen weight; (d) colon length; and (e) typical photographs of colon length in all mice. Data are expressed as the means ± S.E.M. of 8 mice in each group. ^#^*p* < 0.05, ^##^*p* < 0.01 versus normal control (NC) group; ^*∗*^*p* < 0.05, ^*∗∗*^*p* < 0.01 versus DSS group.

After drinking water with 2% DSS for 10 days, the body weight of mice in DSS group was significantly reduced, accompanied by reduced diet, listless air, long urine, soft stools, abdominal distension, and others. However, unlike the sustained body weight loss of mice in DSS group with the prolongation of UC course, the AAC-L/M/H delayed the loss of body weight after intervention. Especially on Day 35, there was no significant difference between the AAC-M/H groups and the positive control (SASP) (*p* > 0.05) (Figure 2(a)).

Disease activity index (DAI) is a comprehensive score for the percentage of weight loss, stool consistency, and stool bleeding to evaluate the severity of UC. The DAI indices of mice in DSS group were observably higher than that of the NC group (*p* < 0.01) (Figure 2(b)). However, the DAI indices of mice in AAC-L/M/H groups were declined significantly on 35 days (*p* < 0.01). The spleen weight increases with the aggravation of UC; hence, spleen hyperplasia is an important pathological feature of UC. On Day 35, the spleen weights of the AAC-L/M/H groups were significantly lower than that of the DSS group (*p* < 0.01) (Figure 2(c)). The colon length shortens with the prolongation of the course of UC, which aggravates the pathological features such as diarrhea and hematochezia. The colon length in DSS group was shortened and was markedly lower than that of the NC group (*p* < 0.01) (Figures 2(d) & 2(e)). After treatment of 35 days, the colon length in the AAC-H group was dramatically longer than that in the DSS group (*p* < 0.01).

### 3.2. Effects of AAC on the Production of Cytokines and MPO

**Figure 3:**
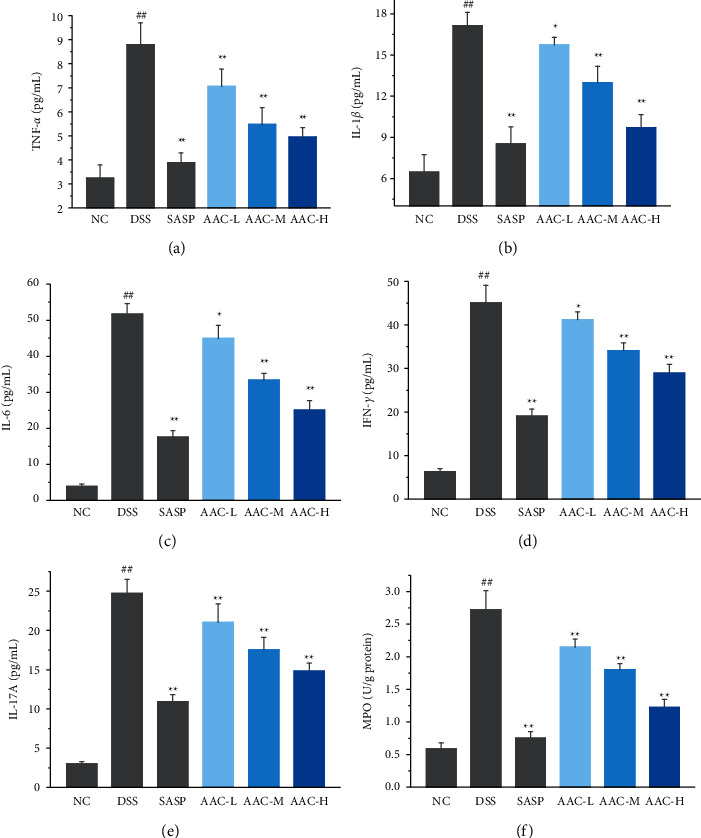
The contents of inflammatory cytokines and MPO determined by ELISA in colon tissues of mice. (a) TNF-*α*; (b) IL-1*β*; (c) IL-6; (d) IFN-*γ*; (e) IL-17A; and (f) MPO. Data are expressed as the means ± S.E.M. of 6 mice in each group. ^#^*p* < 0.05, ^##^*p* < 0.01versus normal control (NC) group; ^*∗*^*p* < 0.05, ^*∗∗*^*p* < 0.01 versus DSS.

The effects of AAC on inflammatory cytokine production and MPO activity were investigated by determining the levels of TNF-*α*, IL-1*β*, IL-6, IFN-*γ*, IL-17A, and MPO in colonic tissues with ELISA kits. Compared with the NC group, the levels of TNF-*α,* IL-1*β*, IFN-*γ*, IL-17 A, IL-6, and MPO in the colon tissue of the DSS group were markedly increased (*p* < 0.01) (Figure 3), whereas the levels of the aforementioned cytokines and MPO were dramatically decreased in AAC-L/M/H groups compared with the DSS group (*p* < 0.01). Meanwhile, the AAC displayed distinct dose-dependent anti-inflammatory effects in AAC-L/M/H groups.

### 3.3. Effects of AAC on TNF-*α*, IL-1*β*, and IL-6 mRNA Expression

**Figure 4:**
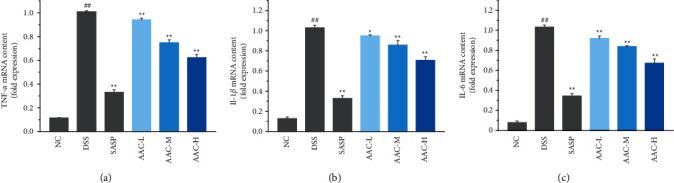
The relative contents of mRNA determined by qRT-PCR in colon tissues of mice. (a) TNF-*α*; (b) IL-1*β*; and (c) IL-6. Data are expressed as the means ± S.E.M. of 6 mice in each group. ^#^*p* < 0.05, ^##^*p* < 0.01 versus normal control (NC) group; ^*∗*^*p* < 0.05, ^*∗∗*^*p* < 0.01 versus DSS group.

The abnormal expression of inflammatory cytokines in colonic mucosa tissue plays an important role in the pathological process of UC, therein IL-1*β*, IL-6, and TNF-*α* are important proinflammatory factors. In this regard, the present results displayed that the DSS treatment significantly increased the mRNA expressions of TNF-*α* (Figure 4(a)), IL-1*β* (Figure 4(b)), and IL-6 (Figure 4(c)) in the colon tissue of the DSS group compared with the NC group (*p* < 0.01). After the AAC intervention, however, the mRNA expressions of TNF-*α*, IL-1*β*, and IL-6 in the colon tissue of AAC-L/M/H groups were notably reduced compared with the DSS group (*p* < 0.01).

### 3.4. Effects of AAC on MAPK, STAT3, and NF-*κ*B Signaling Pathways

**Figure 5:**
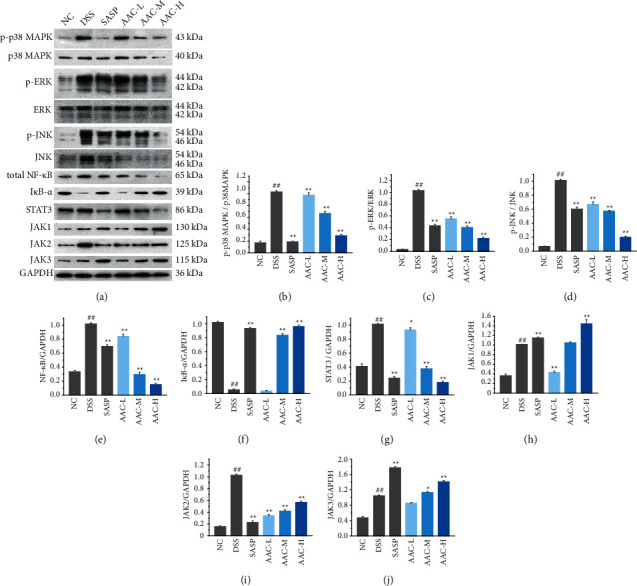
The inhibitory effects of AAC on the activation of MAPK, NF-*κ*B, and STAT3 signaling pathways. The representative protein bands (a) and levels of phosphorylated p38 MAPK/p38 MAPK (b), phosphorylated ERK/ERK (c), phosphorylated JNK/JNK (d), total NF-*κ*B p65 (e), I*κ*B-*α* (f), STAT3 (g), JAK1 (h), JAK2 (i), and JAK3 (j) in colon tissues of mice were analyzed by Western blot analysis. Data are expressed as the means ± S.E.M. of 3 mice in each group. #*p* < 0.05, ##*p* < 0.01 versus normal control (NC) group; ^*∗*^*p* < 0.05, ^*∗∗*^*p* < 0.01 versus DSS group.

In the present study, several key proteins in the MAPK, NF-*κ*B, and STAT3 signaling pathways were detected with Western blot analysis (Figure 5(a)).

Currently, the protein expression of p-p38 MAPK/p38 MAPK (Figure 5(b)) in the colon tissue of the DSS group wassignificantly increased compared with the NC group (*p* < 0.01), while it was memorably suppressed in AAC-M/H groups after AAC treatment (*p* < 0.01). Similarly, the phosphorylation levels of ERK (Figure 5(d)) and JNK (Figure 5(d)) proteins in DSS group were significantly higher than that those in the NC group (*p* < 0.01). Meanwhile, AAC-L/M/H memorably decreased the phosphorylation levels of JNK and ERK proteins in UC mice (*p* < 0.01).

For the NF-*κ*B signaling pathway, Western blot analysis indicated that, compared with the NC group, the protein expressions of NF-*κ*B p65 (Figure 5(e)) and I*κ*B-*α* (Figure 5(f)) of the DSS group were significantly increased (*p* < 0.01) and reduced (*p* < 0.01), respectively. While compared with the DSS group, the above two protein expressions in AAC-L/M/H groups were distinctly reduced (*p* < 0.01) and increased (*p* < 0.01), respectively.

As for the JAK/STATs signaling pathway, compared with the NC group, the protein expressions of STAT3 (Figure 5(g)) and JAK2 (Figure 5(i)) in the DSS group were significantly increased (*p* < 0.01), respectively. While compared with the DSS group, the expressions of JAK2 and STAT3 in the SASP group and ACC-L/M/H groups were significantly reduced (*p* < 0.01), respectively. However, at the current doses, SASP and ACC promoted the expressions of JAK1 (Figure 5(h)) and JAK3 (Figure 5(j)), and ACC displayed a negative regulatory effect on JAK1-JAK3.

### 3.5. Histopathology

**Figure 6:**
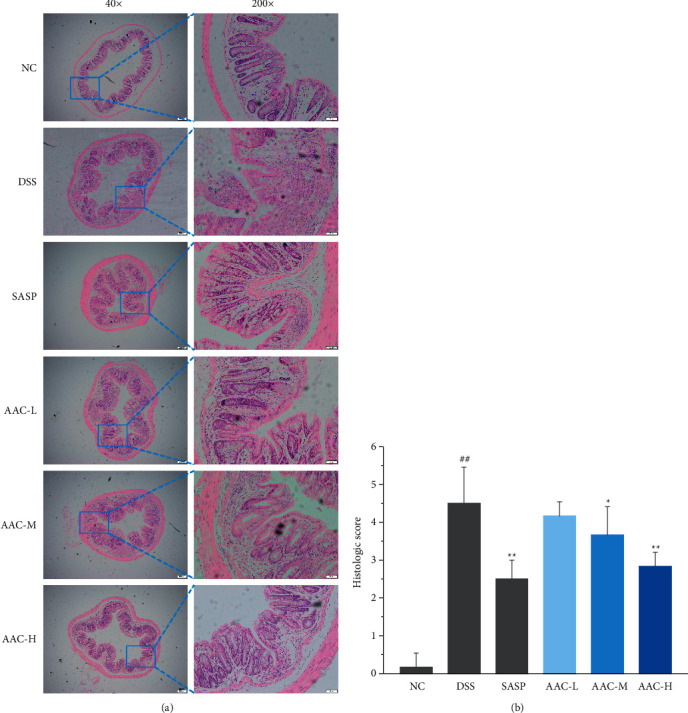
Histological evaluation of colon tissues. (a) The representative images of H&E staining (magnification 40× and 200×) and (b) the corresponding histologic scores. Data are expressed as the means ± S.E.M. of 6 mice in each group. ^#^*p* < 0.05, ^##^*p* < 0.01 versus normal control (NC) group; ^*∗*^*p* < 0.05, ^*∗∗*^*p* < 0.01 versus DSS group.

Histological evaluations of the colon tissues were accomplished with hematoxylin and eosin staining, followed by the microscopic examination. The colon mucosal epithelium of the DSS group was partially defective, with reduced glands and disordered arrangement; mucosal edema and congestion were abundant, and the number of recesses and goblet cells was obviously reduced; a large number of inflammatory cells were infiltrated in the mucosa and submucosa, forming ulcer foci (Figure 6(a)). Histologic scores were significantly higher than those in the NC group (Figure 6(b)) (*p* < 0.01), indicating that the UC model was successfully induced.

After treatment with AAC at concentrations of 3, 6, and 12 g/kg, the colon tissues were intact, and the goblet cells were clearly visible, which was significantly improved compared with the DSS group; there was still a small amount of inflammatory cell infiltration, although the mucosal was repaired to some degrees. Histologic score in the AAC-H group was significantly lower than that in the DSS group (Figure 6(b)) (*p* < 0.01).

### 3.6. Content Analysis of AAC

**Figure 7:**
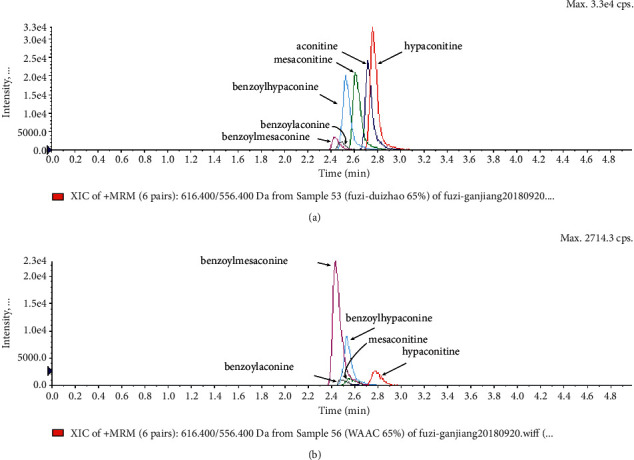
Total ion chromatograms of 6 referential alkaloids (a) and alkaloids in AAC-L (b).

According to the Chinese Pharmacopoeia (2020), six alkaloids, including three diester alkaloids of aconitine, hypaconitine, and mesaconitine and three monoester alkaloids of benzoylaconine, benzoylhypaconine, and benzoylmesaconine, are marked chemical components for quality control of aconiti lateralis radix praeparata. In this regard, the qualitative and quantitative analysis of six alkaloid standards (Figure 7(a)) and AAC-L (Figure 7(b)) were implemented with the LC-MS/MS analysis. Since aconitine was not detected according to the total ion chromatograms (Figure 7(b) and Table 2), its content in AAC-L was marked as 0, and according to the above contents of alkaloids, AAC-M/H were calculated. Obviously, the contents of the three high toxic diester alkaloids were significantly reduced after the processing in this study in which aconitine was extremely low or 0, and hypaconitine and mesaconitine were also markedly reduced.

### 3.7. Molecular Docking Simulations

In the present study, molecular docking was employed to investigate the binding mode between the MAPK/NF-*κ*B/STAT3 enzymes and potential ligands. The BEs, *in silico* half inhibitory concentration (IC_50_) values, and hydrogen bonds (H-bond) are shown in Table 3.

As shown in Table 3, the binding strength order is MAPK > NF-*κ*B > STAT3 by comparing the BEs of six alkaloids with three key enzymes in regarding to the BEs and IC_50_ values. Especially, benzoylhypaconine displayed relatively higher binding strengths and inhibitory activities on MAPK, NF-*κ*B, and STAT3, with BEs values of −7.06 kal/M, −5.31 kal/M, and −4.73 kal/M, and with IC_50_ values of 6.70 *μ*M, 127.31 *μ*M, and 338.44 *μ*M, respectively.

**Figure 8:**
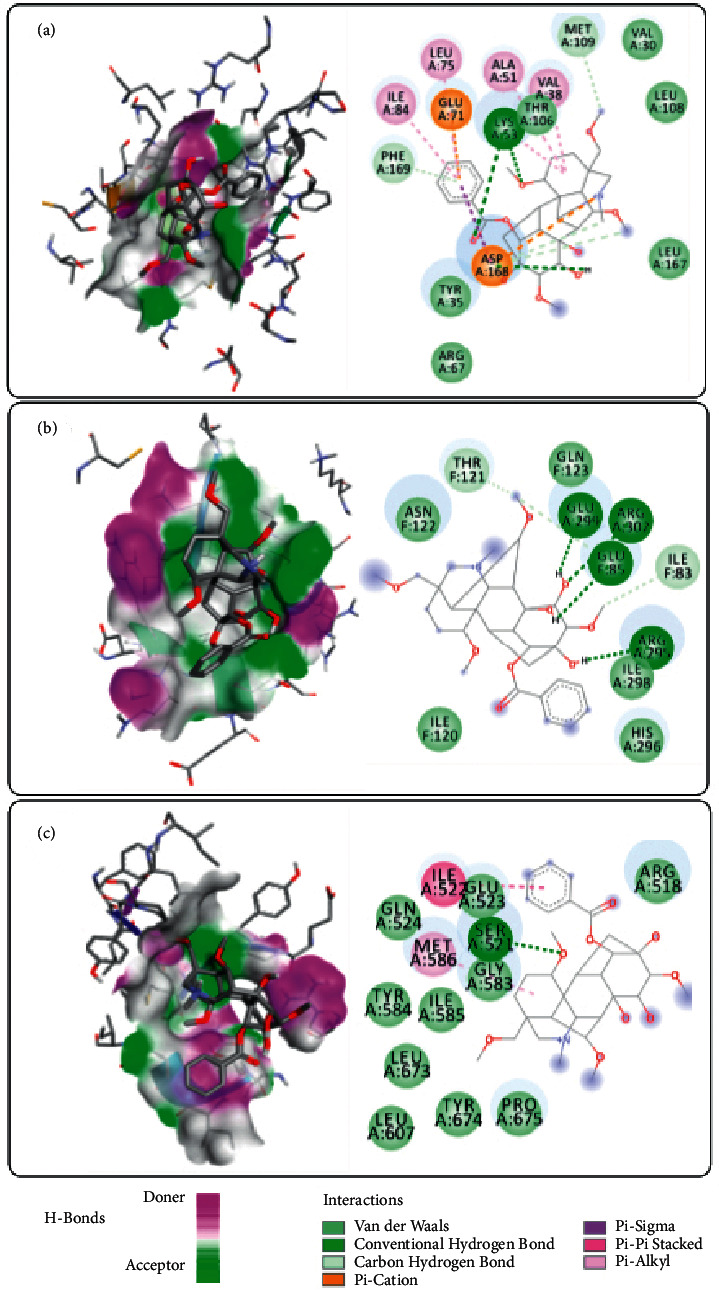
Docking simulations of benzoylhypaconine with MAPK (a), NF-*κ*B (b), and STAT3 (c). Ala, alanine; Arg, arginine; Asp, aspartic acid; Gln, glutamine; Glu, glutamic acid; Gly, glycine; His, histidine; Ile, isoleucine; Leu, leucine; Lys, lysine; Met, methionine; Phe, phenylalanine; Ser, serine; Thr, threonine; Tyr, threonine; Val, valine. H-bond, hydrogen bond.

The three-dimensional docking models of benzoylhypaconine with MAPK, NF-*κ*B, and STAT3 active sites were further simulated (Figure 8). It can be seen that benzoylhypaconine formed H-bonds with the amino acid residues of Lys53, Glu85, Arg295, Glu299, Arg302, and Ser521 to MAPK, NF-*κ*B, and STAT3, respectively. Meanwhile, it kept close contacts, such as van der Waals interaction (E_vdW_), coulomb interaction (E_elec_), and hydrophobic interaction (E_ASA_), with other amino acid residues, such as Val30, Tyr35, Val38, Ala51, Arg67, Glu71, Leu75, Ile84, Tyr106, Leu108, Met109, Leu167, Asp168, Phe169 to MAPK (Figure 8(a)), and Ile83, Ile120, Thr121, Asn122, Gln123, His296, Ile298 to NF-*κ*B (Figure 8(b)), and Arg518, Ile522, Glu523, Gln524, Gly583, Tyr584, Ile585, Leu607, Leu673, Tyr674, Pro675 to STAT3 (Figure 8(c)), respectively.

## 4. Discussion

According to the existing UC treatment drugs, it is unconfident to effectively control UC, a refractory intestinal disease [24]. Therefore, finding new UC therapeutics from natural medicines is becoming a hot research topic in gastroenterology [25]. The DSS-induced UC in mice is currently one of the most widely used experimental UC model of which the clinical symptoms, pathological manifestations, and lesions are similar to human UC, such as typical weight loss, colon shortening, thickening of intestinal wall, diarrhea, and bloody stools [26]. Although we had confirmed in previous studies [22] that aconiti lateralis radix praeparata (the seminal root of *Aconitum carmichaelii* Debx) can effectively inhibit UC by inhibiting MAPK, NF-*κ*B, and STAT3 signaling pathways, the types of components in *A. carmichaelii* that exert anti-UC therapy remain unclear.

In present study, freely drinking DSS solution caused the corresponding clinical symptoms of UC in mice, indicating that they were successfully modeled, whereas, after AAC intervention, the aforementioned indicators of AAC-L/M/H groups were significantly different from those in DSS group with noteworthy dose-dependent relationship (Figure 2). In addition, according to the pathological examination, AAC treatment notably relieved the pathological symptoms of UC (Figure 6(a)) and decreased the pathological score (Figure 6(b)). Hence, the above results indicated that AAC effectively improved the weight loss, spleen hyperplasia and colon shortening, and repaired the colon tissue, thereby alleviating the symptoms of UC in colitis mice.

The occurrence of UC is closely related to the imbalance of inflammatory factors and abnormal immune response. In UC patients, the levels of pro-inflammatory cytokines in the colon are increased and the levels of anti-inflammatory cytokines are decreased, resulting in immune dysfunction [27]. During the intestinal inflammation, the abnormally elevated secretion levels of pro-inflammatory cytokines, such as TNF-*α*, IL-1*β*, and IL-6, induce the apoptosis of intestinal epithelial cells with autophagy deficiency and decrease the intestinal shielding function, which is one of the main reasons for the inflammatory injury of colon tissue [28]. Therefore, the inflammatory response of UC can be reduced by inhibiting the release of pro-inflammatory cytokines.

In the present study, the levels of TNF-*α* (Figure 3(a)), IL-1*β* (Figure 3(b)), and IL-6 (Figure 3(c)) in the colon tissue were markedly reduced after AAC treatment, suggesting that AAC could inhibit intestinal inflammation and repair the inflammatory injury. Moreover, the mRNA expressions of TNF-*α* (Figure 4(a)), IL-1*β* (Figure 4(b)), and IL-6 (Figure 4(c)) in colonic tissues of AAC-L/M/H groups were also notably downregulated after AAC intervention, which suggested that AAC suppressed the release of TNF-*α*, IL-1*β,* and IL-6 in colon tissue by inhibiting the mRNA overexpression and thus is of great significance for the current clinical prevention and treatment of UC [27, 28].

IFN-*γ* stimulates Th17 cells to produce IL-17A and other cytokines, prompting them to disrupt the intestinal mucosal immune balance, therein IL-17 A further stimulates the release of TNF-*α* and chemokines [29]. MPO is a lysosomal protein mainly found in neutrophils and is one of the most important biomarkers of neutrophil infiltration and acute inflammation [30]. Thus, the decrease of MPO activity in colon tissue represents the inhibition of neutrophil accumulation [31]. Presently, the levels of IFN-*γ* (Figure 3(d)), IL-17 A (Figure 3(e)), and MPO activity (Figure 3(f)) were dramatically decreased in AAC-L/M/H groups, suggesting that AAC not only repaired the mucosal barrier by inhibiting the expression of IFN-*γ* and IL-17a but also reduced the neutrophil infiltration and acute inflammation by inhibiting the accumulation of MPO in the colon tissue, thereby alleviating the clinical symptoms of UC mice.

MAPK signaling pathway is involved in the whole process of UC, and inhibition of which will observably reduce the inflammation and apoptosis caused by UC [32]. As a member of the MAPK family, the activated p38 MAPK pathway promotes the secretion of intracellular cytokines, such as TNF-*α* and IL-1*β*, to mediate the occurrence of inflammation, while in return these cytokines activate the p38 MAPK signaling pathway through paracrine action, forming a vicious cycle during the inflammatory response [33]. Meanwhile, JNK and ERK signaling pathways regulate the expression and secretion of inflammatory cytokines, such as IL-6 and TNF-*α*. That is to say, inactivation of JNK and ERK signaling pathway will markedly reduce the secretion of inflammatory cytokines and reduce colon injury [34, 35]. In the present study, the protein phosphorylation levels of p38 MAPK (Figure 5(b)), JNK (Figure 5(c)), and ERK (Figure 5(d)) were memorably suppressed in colon tissue of AAC-M/H groups after treatment, indicating that AAC successfully inhibited the phosphorylation of p38 MAPK, JNK, and ERK signaling pathways, so as to avoid the occurrence of inflammatory factor storms.

Activation of NF-*κ*B is a central link in the inflammatory response. In the resting state, NF-*κ*B (p50/p65) binds to its inhibitory protein I*κ*B*α* to form a trimer, which stably exists in the cytoplasm and inhibits the transfer of NF-*κ*B to the nucleus [36]. When stimulated by inflammation, I*κ*B*α* is degraded by phosphorylation or ubiquitination, resulting in its dissociation from the trimer. Then, the NF-*κ*B dimer exposes its nuclear localization sequence and quickly migrates from the cytoplasm to the nucleus. When combined with specific sequences on nuclear DNA, it upregulates the mRNA expressions of TNF-*α*, IL-6, and IL-1*β*, which further activates the NF-*κ*B signaling pathway and causes inflammatory cascade reaction, leading to the intestinal inflammatory response and mucosal injury [37]. The present results displayed the protein expressions of NF-*κ*B p65 (Figure 5(e)) and I*κ*B*α* (Figure 5(f)) in AAC-L/M/H groups were distinctly reduced and increased, respectively. Therefore, it was speculated that ACC inhibited the degradation of I*κ*B*α* to impede the entry of NF-*κ*B into the nucleus, thereby improving UC.

The JAK/STATs signaling pathway is a series of multi-effect cascade signal transduction pathways stimulated by cytokines, which mediates the occurrence and development of UC [38]. JAK2 and STAT3 are key regulators in the JAK/STAT signaling pathway [39], wherein JAK2 phosphorylates downstream STAT3 after recognizing extracellular signals; then, p-STAT3 regulates the expression of multiple downstream genes and participate in the regulation of apoptosis and inflammation [39, 40]. Presently, AAC significantly downregulated the protein expressions of STAT3 (Figure 5(g)) and JAK2 (Figure 5(i)) in the colon tissue of ACC-L/M/H groups. However, ACC at the current doses failed to suppress the expressions of JAK1 (Figure 5(h)) and JAK3 (Figure 5(j)), and displayed a negative regulatory effect on JAK1-JAK3. Hence, the present results suggested that ACC suppressed the regulation of the JAK2/STAT3 signaling pathway in the nucleus by inhibiting the phosphorylation of JAK2 and STAT3.

MAPK, NF-*κ*B, and STAT3 signal pathways interact mutually in the pathogenesis of UC and synergistically promote the progress of UC. Activated p38 MAPK promotes degradation of I*κ*B through phosphorylation and ubiquitination, which accelerate NF-*κ*B entering the nucleus and generate multiple inflammatory cytokines, which in turn activate MAPK signaling pathways, thereby amplifying inflammatory responses [41]. Besides, NF-*κ*B and STAT3 are extensively involved in many physiological processes such as immunity, inflammation, metabolism, and cell differentiation [42]. Our study confirmed that AAC comprehensively inhibited the activation of the above three signaling pathways by downregulating the phosphorylation of p38 MAPK, ERK, and JNK, as well as the production of NF-*κ*B and STAT3 and inhibiting the degradation of I*κ*B.

According to the Chinese Pharmacopoeia, the six alkaloids such as aconitine, hypaconitine, mesaconitine, benzoylaconine, benzoylhypaconine, and benzoylmesaconine are the marked chemical components for quality control of aconiti lateralis radix praeparata. In clinical, these alkaloids are the main medicinal and toxic components, which hence make *A. carmichaelii* a medicine with a narrow therapeutic window [43]. In other words, high alkaloid contents of *A. carmichaelii* cause poisoning, which may lead to death in severe cases [44, 45]. The HPLC-MS/MS analysis indicated that the three high toxic diester alkaloids were significantly reduced after the processing in this study. It is interesting that the three less toxic monoester alkaloids were higher in both contents and proportion than that of the three high toxic diester alkaloids. That is to say, after processing in this study, the contents of toxic alkaloids, especially the contents of the three high toxic alkaloids, were significantly reduced, thus greatly reduced the toxicity of *A. carmichaelii*. In addition, although the contents of the three low toxic monoester alkaloids were decreased compared with *A. carmichaelii* decoction in our previous study, their proportion were increased, which on the other hand ensured its anti-UC efficacy. To this point, structure-activity relationship studies also showed that the high toxic diester alkaloids are hydrolyzed and changed in structures to generate low toxic monoester benzoylaconine, thereby reducing toxicity in the process of decocting [46].

Molecular docking predicts the optimum binding mode and reviews the interactions between the acceptor-ligand complexes by studying their binding energy (BE), action sites, and key residues. Hence, it is an important approach to predict and study the interaction pattern between small molecule ligands and protein receptors, and has become a widely used method in drug design [47]. Presently, from the perspective of structure types in Table 3, the BEs of the three monoester alkaloids with MAPK, NF-*κ*B, and STAT3 were generally lower than that of the 3 diester alkaloids, and the IC_50_ values were generally smaller, indicating that the three monoester alkaloids possessed higher affinity and inhibitory activities on the three key enzymes. Therein, benzoylhypaconine displayed relatively higher binding strengths and inhibitory activities on MAPK, NF-*κ*B, and STAT3. In this regard, the molecular docking method provides a convenient method to explore the interaction mechanisms of MAPK, NF-*κ*B, and STAT3 with potential ligands in *A. carmichaelii* and is of considerable reference for the development of bioactive components from *A. carmichaelii* with anti-UC activities.

## 5. Conclusions

In the present study, DSS-induced UC model was established to study the anti-UC effect of AAC on colitis mice. The results suggested that AAC effectively improved the weight loss, DAI, spleen hyperplasia, and colon shortening, and thus alleviated the symptoms of colitis mice. In addition, AAC not only inhibited the MPO enzyme and the abnormal secretion of inflammatory cytokines (TNF-*α*, IL-1*β*, IL-6, IFN-*γ*, and IL-17A) and suppressed the overexpression of inflammatory mediators (TNF-*α*, IL-1*β*, and IL-6) mRNA but also reduced the phosphorylation of p38 MAPK, ERK, and JNK, and the protein expressions of NF-*κ*B, I*κ*B-*α*, STAT3, and JAK2 in the colon tissue. Molecular docking results displayed that the six alkaloids in *A. carmichaelii*, especially the three monoester alkaloids, exhibited higher binding affinities with the key target proteins of MAPK, NF-*κ*B, and STAT3. In conclusion, the present study indicated that the anti-UC effect of AAC might be highly related to the inhibition of MAPK/NF-*κ*B/STAT3 signaling pathway, which would further provide practical and edge-cutting background information for the development and utilization of *A. carmichaelii* as a potential natural anti-UC remedy.

## Figures and Tables

**Table 1:** Oligonucleotide sequence in quantitative real-time PCR.

Gene	Primer	Sequences (5′–3′)
TNF-*α*	Forward	5′-TCT ACT CCC AGG TTC TCT TCA-3′
	Reverse	5′-CCT GGT ATG AGA TAG CAA ATC G-3′

IL-1*β*	Forward	5′-GCA GGC AGT ATC ACT CAT TGT-3′
	Reverse	5′-GGA AAA GAA GGT GCT CAT GT-3′

IL-6	Forward	5′-ACT TCC ATC CAG TTG CCT TCT TGG-3′
	Reverse	5′-TTA AGC CTC CGA CTT GTG AAG TGG-3′

*β*-actin	Forward	5′-GCT TCT AGG CGG ACT GTT AC-3′
	Reverse	5′-CCA TGC CAA TGT TGT CTC TT-3′

**Table 2:** Concentrations of alkaloids in AAC solutions (*μ*g/mL).

	AAC-L	AAC-M	AAC-H
Aconitine	Undetected	0	0
Hypaconitine	11.4	22.8	45.6
Mesaconitine	8.29	16.58	33.16
Benzoylaconine	64.2	128.4	256.8
Benzoylhypaconine	34.9	69.8	139.6
Benzoylmesaconine	852	1704	3408

**Table 3:** Molecular docking simulations between six alkaloids and MAPK/NF-*κ*B/STAT3.

No.	Ligand	Chemical structure	Length (A)	MAPK			NF-*κ*B			STAT3		
				BE (kal/M)	H-bond atoms	IC_50_ (*μ*M)	BE (kal/M)	H-bond atoms	IC_50_ (*μ*M)	BE (kal/M)	H-bond atoms	IC_50_ (*μ*M)
1	Aconitine	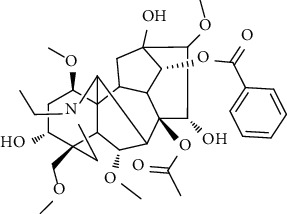	12.7	−5.92	Lys53	45.66	−4.07	Arg295	1070	−2.34	Glu523	19360
					Met109							

2	Hypaconitine	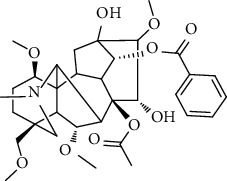	11.5	−6.42	Tyr35	19.57	−4.63	Tyr195	400.52	−3.56	Arg518	2520
					Lys53			Arg295				

3	Mesaconitine	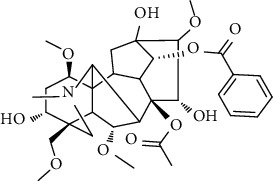	11.4	−6.23	Met109	27.09	−4.42	Glu299	574.10	−3.52	Arg518	2610
					Asp168			Arg302				

4	Benzoylaconine	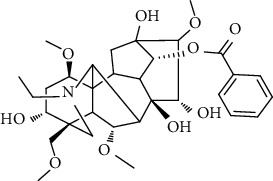	11.3	−6.04	Lys53	37.33	−4.40	Glu85	595.91	−3.64	Ser509	2150
								Arg295			Trp510	
					Met109			Glu299			Ser513	
					Asp168			Arg302			Leu520	

5	Benzoylhypaconine	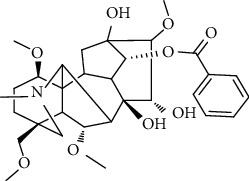	12.0	−7.06	Lys53 (×2)	6.70	−5.31	Glu85	127.31	−4.73	Ser521	338.44
								Arg295				
								Glu299				
								Arg302				

6	Benzoylmesaconine	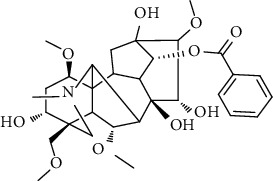	10.9	−6.11	Val30	33.14	−4.89	Arg295	261.38	−3.78	Ser521	1700
					Met109							
					Asp168			Glu299			Gly583	

## Data Availability

The datasets used during the current study are available from the corresponding author on reasonable request.

## Abbreviations

5-ASA: 5-Aminosalicylic acid
Ala: Alanine
Arg: Arginine
ANOVA: Analysis of variance
Asp: Aspartic acid
BCA: Bicinchoninic acid
cDNA: Complementary deoxyribonucleic acid
DAI: Disease activity index
DSS: Dextran sulfate sodium
ELISA: Enzyme-linked immunosorbent assay
ERK: Extracellular regulating kinase
GAPDH: Glyceraldehyde-3-phosphate dehydrogenase
Gln: Glutamine
Glu: Glutamic acid
Gly: Glycine
His: Histidine
H&E: Hematoxylin and eosin
I*κ*B: Inhibitor kappa B
IBD: Inflammatory bowel disease
IFN-*γ*: Interferon-*γ*
IL: Interleukin
JAK: Janus-activated kinase
JNK: C-Jun N-terminal kinases
Leu: Leucine
Lys: Lysine
MAPK: Mitogen-activated protein kinase
Met: Methionine
MPO: Myeloperoxidase
MPO: Myeloperoxidase
NC: Normal control
NF-*κ*B: Nuclear factor-kappa B
NO: Nitric oxide
RNA: Ribonucleic acid
S.E.M.: Standard error of mean
SASP: Sulfasalazine
SDS-PAGE: Sodium dodecyl sulfate-polyacrylamide gel electrophoresis
STAT3: Signal transducer and activator of transcription 3
Ser: Serine
TCM: Traditional Chinese medicine
Th17: T helper 17
TNF: Tumor necrosis factor
Treg: Regulatory T
Trp: Tryptophan
Tyr: Threonine
Val: Valine
BE: Binding energy
H-bond: Hydrogen bond
IC_50_: Estimated half inhibition concentration (i*n silico*)
UC: Ulcerative colitis.

## Supplementary Materials

Supplementary Materials The PDF file *Figures-other 5 alkaloids molecular docking* contains molecular docking simulations among aconitine, hypaconitine, mesaconitine, benzoylaconine, benzoylmesaconine, and MAPK/NF-*κ*B/STAT3 proteins. The PDF file *Change of body weight & DAI & Colon Length & Spleen Weight* contains the clinical data of UC mice. The JPG files *WB1* and *WB2* are representative WB gel bands existing in this article.
