# Supplementary material for: Alkaloids from Aconitum carmichaelii Alleviates DSS-Induced Ulcerative Colitis in Mice via MAPK/NF-κB/STAT3 Signaling Inhibition
Source: Evid Based Complement Alternat Med. 2022 May 31;2022:6257778. doi: 10.1155/2022/6257778 (PMC9173982; doi:10.1155/2022/6257778)
Supplement: Supplementary Materials — The PDF file Figures-other 5 alkaloids molecular docking contains molecular docking simulations among aconitine, hypaconitine, mesaconitine, benzoylaconine, benzoylmesaconine, and MAPK/NF-κB/STAT3 proteins. The PDF file Change of body weight & DAI & Colon Length & Spleen Weight contains the clinical data of UC mice. The JPG files WB1 and WB2 are representative WB gel bands existing in this article. [file 6257778.f1.zip › 6257778.f1/Change of body weight & DAI & Colon Length & Spleen Weight - upload.pdf]

Day 0	NC	DSS	SASP	AAC-L	AAC-M	AAC-H
	21.4	23.2	21.2	22.3	21.9	22.3
	22.2	22.1	22.3	22.7	22.3	21.6
	22	21.3	21.6	22.6	22.2	23.3
	21.8	21.8	22.1	21.2	21.6	22.4
	23.1	22	22.7	22	23.1	21.6
	21.9	21.9	21.9	21.2	22.1	21.6
mean	22.066667	22.05	21.966667	22	22.2	22.133333

Day 5	NC	DSS	SASP	AAC-L	AAC-M	AAC-H
	21.9	23.4	21.6	22.7	22.8	21.3
	22.3	22.5	22.6	23	22.3	22.4
	22.3	21.9	22	23	23.2	23
	22	22.1	22.5	21.4	21.8	22.9
	23	22.1	22.9	22.5	22.8	22.6
	22.2	21.7	22.4	21.3	22	22.7
mean	22.283333	22.283333	22.333333	22.316667	22.483333	22.483333

Day 10	NC	DSS	SASP	AAC-L	AAC-M	AAC-H
	21.9	22.9	22.1	21.6	22.8	21.8
	22.4	22.7	22	22.1	22.9	22.3
	22.5	21.4	21.3	22.9	23	23.4
	22.9	20.7	22.8	22.3	21.6	22.1
	23.3	22.1	22.8	23.7	22.2	22.9
	23.7	22	22.4	21.4	21.7	22
mean	22.783333	21.966667	22.233333	22.333333	22.366667	22.416667

Day 15	NC	DSS	SASP	AAC-L	AAC-M	AAC-H
	22.1	22.2	22.2	21.5	22.5	21.3
	22.4	22.9	22	22	22.4	23
	22.6	21.4	21.6	22.5	22.5	21.8
	22.4	20.8	22	22	21.5	22.2
	23.3	22.5	22.1	22.9	22.1	22.5
	23.2	22	22.2	21.3	22	21.8
mean	22.666667	21.966667	22.016667	22.033333	22.166667	22.1

Day 20	NC	DSS	SASP	AAC-L	AAC-M	AAC-H
	22.2	21.5	22	21.7	22.3	21
	22.4	22	21.7	22	22.3	21.3
	22.7	20.8	21.6	22.2	22.2	22.6
	22.8	20.2	22.1	22	21.1	21.1
	23.5	21.9	22	22.2	21.6	21.8
	23.5	21.3	22.2	21.6	21.6	21.9

mean	22.85	21.283333	21.933333	21.95	21.85	21.616667

Day 25	NC	DSS	SASP	AAC-L	AAC-M	AAC-H
	22.3	21.2	21.3	21.1	22.1	21.1
	22.5	20.8	21.6	21.2	21.6	21
	23	20.2	21.3	21.7	21.7	22.2
	23	20	21.7	21.1	20.9	21.2
	23.2	21.3	21.6	21.6	21.1	21.5
	23.6	21.2	21.2	21.3	21.2	21.5
mean	22.933333	20.783333	21.45	21.333333	21.433333	21.416667

Day 30	NC	DSS	SASP	AAC-L	AAC-M	AAC-H
	22.6	19.4	21.2	20.5	21.6	20.9
	22.7	19.9	21.2	20.4	21.4	20.7
	23.1	19.3	20.7	21	21.2	21.7
	22.8	19.7	21.2	20.3	20.4	21.2
	23.2	20.9	21.3	21	20.6	21.1
	23.9	21	21	20.4	20.8	20.9
mean	23.05	20.033333	21.1	20.6	21	21.083333

Day 35	NC	DSS	SASP	AAC-L	AAC-M	AAC-H
	22.6	18.6	21	20.1	21.3	20.3
	22.6	19.3	20.7	19.6	21	20.4
	23.3	18.4	20.4	20.5	20.6	21.5
	23.2	19	20.6	20	19.9	20.9
	23.5	20.1	20.4	20.4	20.3	20.6
	23.8	20.3	20.3	19.7	20.6	20.5
mean	23.166667	19.283333	20.566667	20.05	20.616667	20.7

体重改变百分比

Day 0	NC	DSS
	1	1
	1	1
	1	1
	1	1
	1	1
	1	1
mean	1	1

Day 5	NC	DSS
	1.023364	1.008621
	1.004505	1.0181
	1.013636	1.028169
	1.009174	1.013761
	0.995671	1.004545
	1.013699	0.990868
mean	1.010008	1.010677

Day 10	NC	DSS
	1.023364	0.987069
	1.009009	1.027149
	1.022727	1.004695
	1.050459	0.949541
	1.008658	1.004545
	1.082192	1.004566
mean	1.032735	0.996261

Day 15	NC	DSS
	1.03271	0.956897
	1.009009	1.036199
	1.027273	1.004695
	1.027523	0.954128
	1.008658	1.022727
	1.059361	1.004566
mean	1.027422	0.996535

Day 20	NC	DSS
	1.037383	0.926724
	1.009009	0.995475
	1.031818	0.976526
	1.045872	0.926606
	1.017316	0.995455
	1.073059	0.972603

mean	1.035743	0.965565

Day 25	NC	DSS
	1.042056	0.913793
	1.013514	0.941176
	1.045455	0.948357
	1.055046	0.917431
	1.004329	0.968182
	1.077626	0.968037
mean	1.039671	0.942829

Day 30	NC	DSS
	1.056075	0.836207
	1.022523	0.900452
	1.05	0.906103
	1.045872	0.90367
	1.004329	0.95
	1.091324	0.958904
mean	1.04502	0.909223

Day 35	NC	DSS
	1.056075	0.801724
	1.018018	0.873303
	1.059091	0.86385
	1.06422	0.87156
	1.017316	0.913636
	1.086758	0.926941
mean	1.050246	0.875169

SASP	AAC-L	AAC-M	AAC-H
1	1	1	1
1	1	1	1
1	1	1	1
1	1	1	1
1	1	1	1
1	1	1	1
1	1	1	1

SASP	AAC-L	AAC-M	AAC-H
1.018868	1.017937	1.041096	0.955157
1.013453	1.013216	1	1.037037
1.018519	1.017699	1.045045	0.987124
1.0181	1.009434	1.009259	1.022321
1.008811	1.022727	0.987013	1.046296
1.022831	1.004717	0.995475	1.050926
1.016763	1.014288	1.012981	1.016477

SASP	AAC-L	AAC-M	AAC-H
1.042453	0.96861	1.041096	0.977578
0.986547	0.973568	1.026906	1.032407
0.986111	1.013274	1.036036	1.004292
1.031674	1.051887	1	0.986607
1.004405	1.077273	0.961039	1.060185
1.022831	1.009434	0.9819	1.018519
1.012337	1.015674	1.00783	1.013265

SASP	AAC-L	AAC-M	AAC-H
1.04717	0.964126	1.027397	0.955157
0.986547	0.969163	1.004484	1.064815
1	0.995575	1.013514	0.935622
0.995475	1.037736	0.99537	0.991071
0.973568	1.040909	0.95671	1.041667
1.013699	1.004717	0.995475	1.009259
1.002743	1.002038	0.998825	0.999599

SASP	AAC-L	AAC-M	AAC-H
1.037736	0.973094	1.018265	0.941704
0.973094	0.969163	1	0.986111
1	0.982301	1	0.969957
1	1.037736	0.976852	0.941964
0.969163	1.009091	0.935065	1.009259
1.013699	1.018868	0.977376	1.013889

0.998949	0.998375	0.984593	0.977147

SASP	AAC-L	AAC-M	AAC-H
1.004717	0.946188	1.009132	0.946188
0.96861	0.933921	0.96861	0.972222
0.986111	0.960177	0.977477	0.95279
0.9819	0.995283	0.967593	0.946429
0.951542	0.981818	0.91342	0.99537
0.968037	1.004717	0.959276	0.99537
0.976819	0.970351	0.965918	0.968062

SASP	AAC-L	AAC-M	AAC-H
1	0.919283	0.986301	0.93722
0.950673	0.898678	0.959641	0.958333
0.958333	0.929204	0.954955	0.93133
0.959276	0.957547	0.944444	0.946429
0.938326	0.954545	0.891775	0.976852
0.958904	0.962264	0.941176	0.967593
0.960919	0.93692	0.946382	0.952959

SASP	AAC-L	AAC-M	AAC-H
0.990566	0.901345	0.972603	0.910314
0.928251	0.863436	0.941704	0.944444
0.944444	0.90708	0.927928	0.922747
0.932127	0.943396	0.921296	0.933036
0.898678	0.927273	0.878788	0.953704
0.926941	0.929245	0.932127	0.949074
0.936835	0.911963	0.929074	0.935553

Day 36	NC	DSS	SASP	AAC-L	AAC-M	AAC-H
	0	3.67	1.33	2.33	1.67	2.33
	0.33	3.67	1.67	2	2.67	1.33
	0	3	1.33	1.33	1.33	1.33
	0	3	1	2.67	2.33	1.33
	0.33	3.67	1.33	2.67	1.33	2
	0	3	1.67	3	2.33	2.33
mean	0.11	3.335	1.3883333	2.3333333	1.9433333	1.775
SD	0.1704113	0.3669741	0.252857	0.5981527	0.5751753	0.5021454

0.00	0
0.33	1
0.67	2
1.00	3
1.33	4
1.67	5
2.00	6
2.33	7
2.67	8
3.00	9
3.33	10
3.67	11
4.00	12

Day 36	NC	DSS	SASP	AAC-L	AAC-M	AAC-H
单位：mm	78	66	68	56	65	69
	76	64	68	63	70	65
	83	57	73	61	66	67
	85	58	70	59	58	71
	77	63	76	60	64	68
	73	60	75	66	67	69
mean	78.666667	61.333333	71.666667	60.833333	65	68.166667
	4.5018515	3.5590261	3.5023801	3.4302575	4	2.0412415

Day 36	NC	DSS	SASP	AAC-L	AAC-M	AAC-H
	0.1042	0.2654	0.1282	0.1944	0.1954	0.1528
	0.1371	0.2417	0.1343	0.2051	0.1837	0.1439
	0.1141	0.2812	0.1105	0.2043	0.2076	0.1766
	0.0987	0.2563	0.1371	0.2163	0.1808	0.1472
	0.1023	0.2848	0.1225	0.2432	0.1686	0.1537
	0.1159	0.2501	0.1279	0.2256	0.1827	0.1404
mean	0.11205	0.26325	0.12675	0.2148167	0.1864667	0.1524333
	0.0140102	0.0171864	0.0094834	0.0175838	0.0134134	0.0128901
