# Supplementary material for: Alkaloids from Aconitum carmichaelii Alleviates DSS-Induced Ulcerative Colitis in Mice via MAPK/NF-κB/STAT3 Signaling Inhibition
Source: Evid Based Complement Alternat Med. 2022 May 31;2022:6257778. doi: 10.1155/2022/6257778 (PMC9173982; doi:10.1155/2022/6257778)

Docking simulations of Aconitine with MAPK (D), NF-κB (E) and STAT3 (F)

MAPK (D)

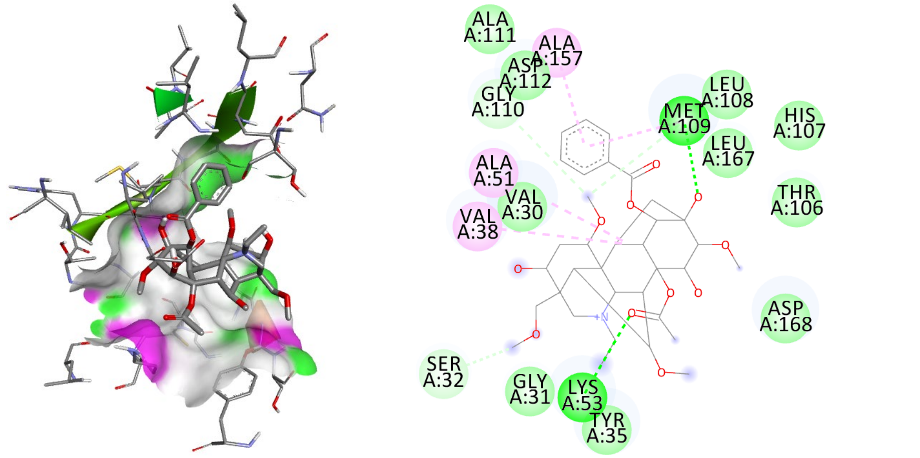

NF-κB (E)

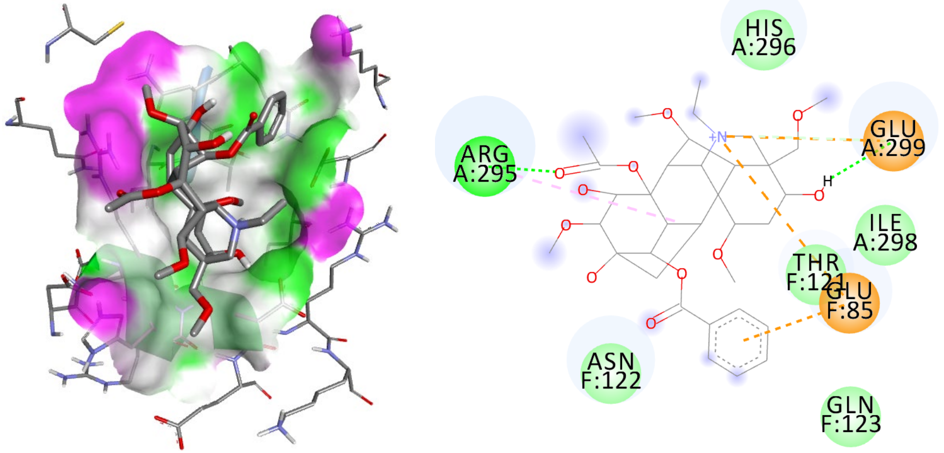

STAT3 (F)

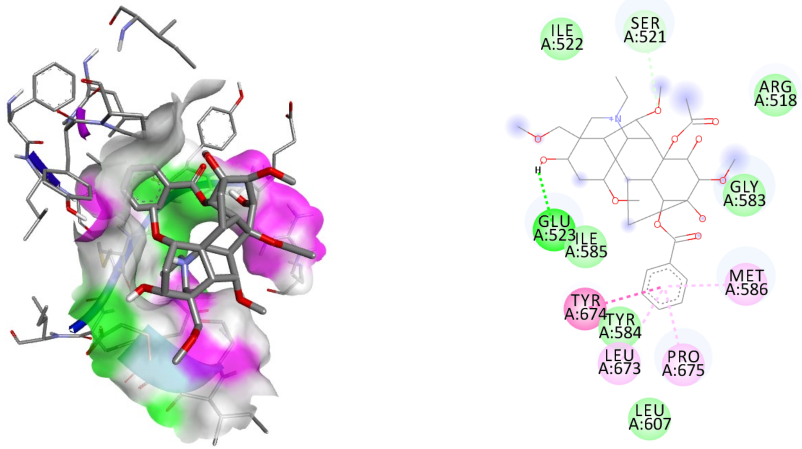

Docking simulations of Hypaconitine with MAPK (G), NF-κB (H) and STAT3 (I)

MAPK (G)

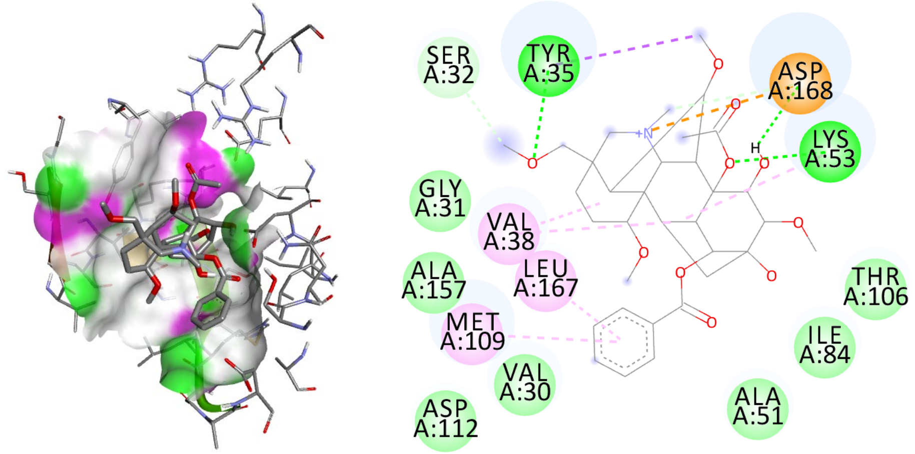

NF-κB (H)

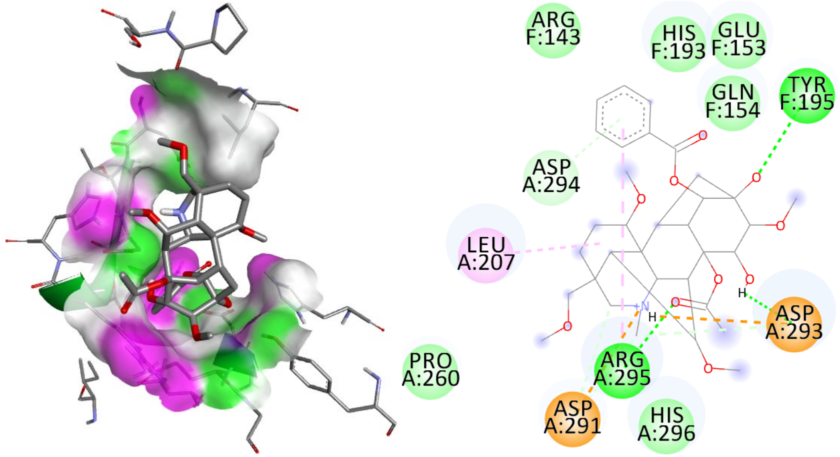

STAT3 (I)

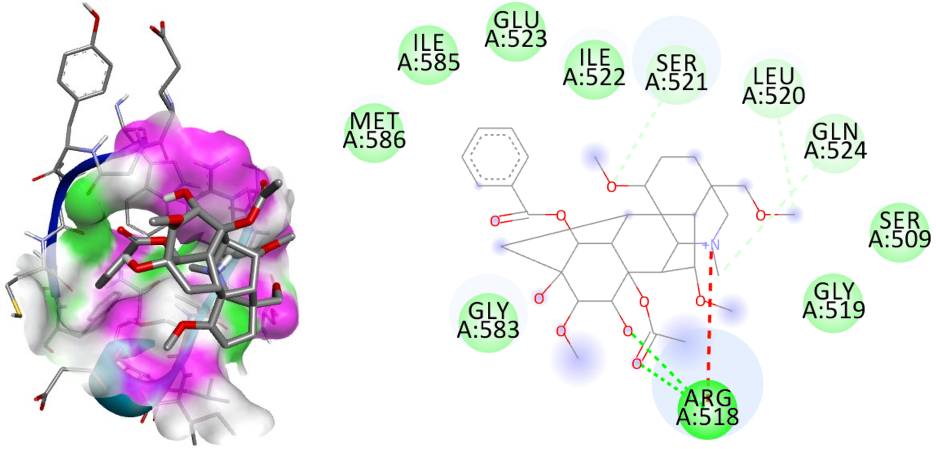

Docking simulations of Mesaconitine with MAPK (J), NF-κB (K) and STAT3 (L)

MAPK (J)

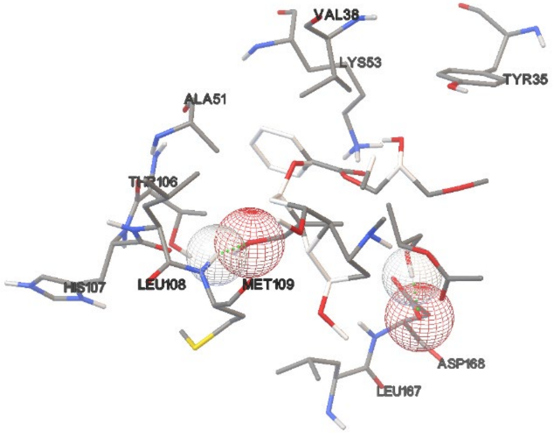

NF-κB (K)

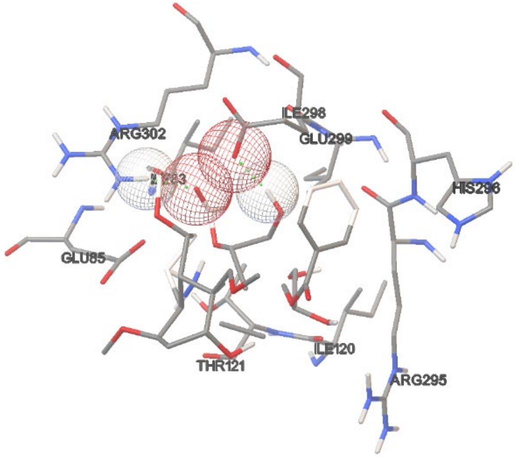

STAT3 (L)

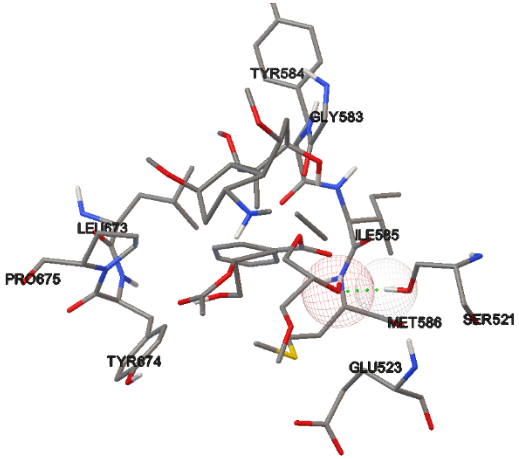

Docking simulations of Benzoylaconine with MAPK (M), NF-κB (N) and STAT3 (O)

MAPK (M)

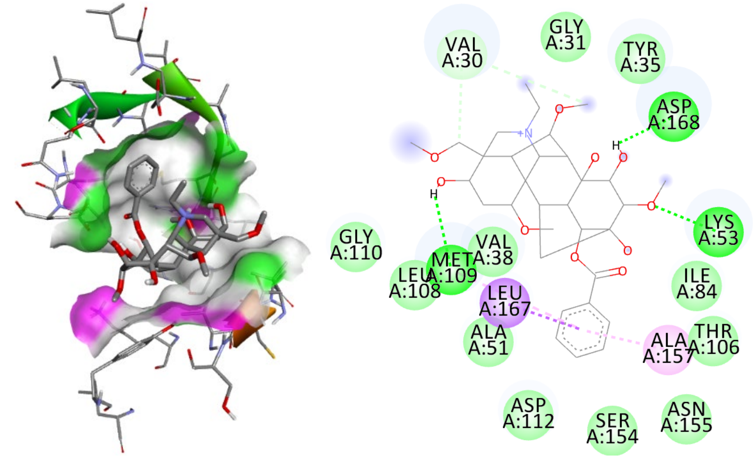

NF-κB (N)

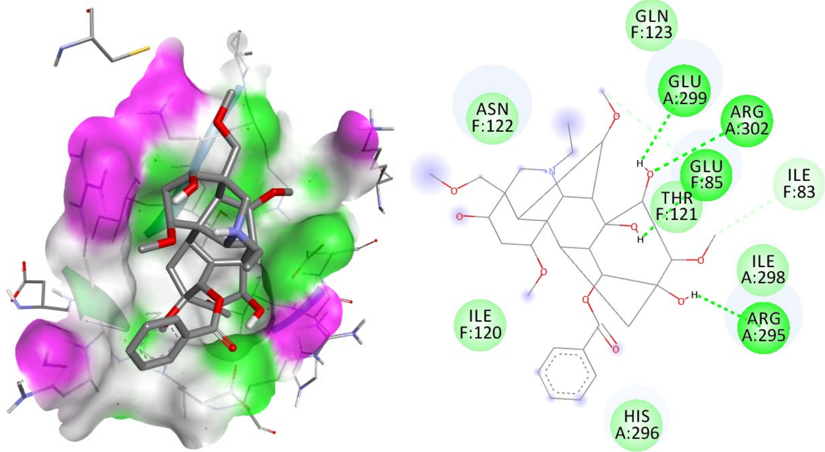

STAT3 (O)

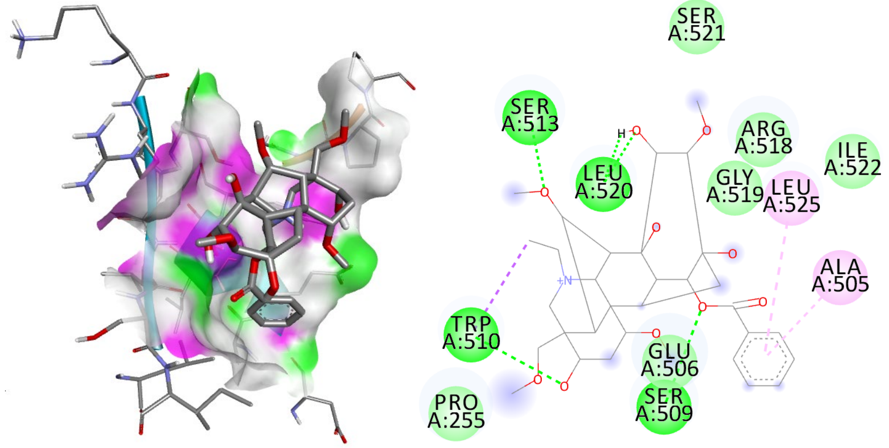

Docking simulations of Benzoylmesaconine with MAPK (P), NF-κB (Q) and STAT3 (R)

MAPK (P)

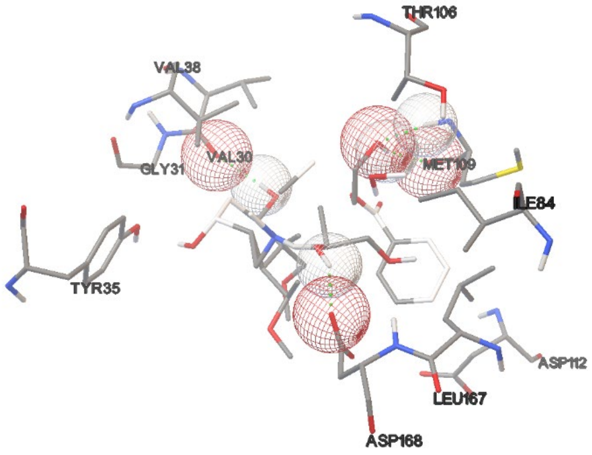

NF-κB (Q)

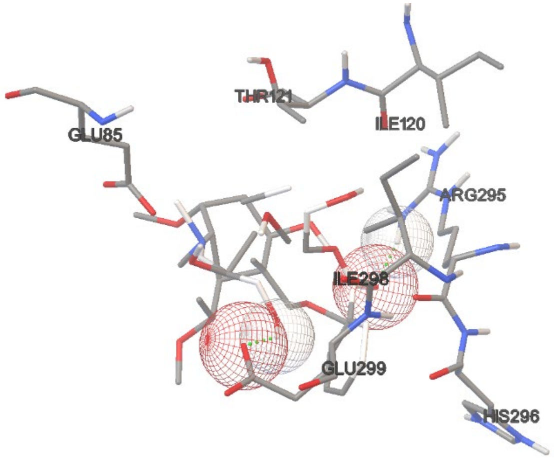

STAT3 (R)

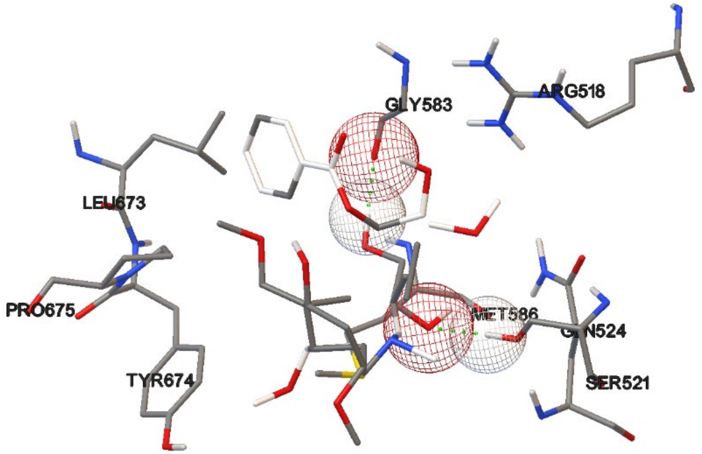

Supplement: Supplementary Materials — The PDF file Figures-other 5 alkaloids molecular docking contains molecular docking simulations among aconitine, hypaconitine, mesaconitine, benzoylaconine, benzoylmesaconine, and MAPK/NF-κB/STAT3 proteins. The PDF file Change of body weight & DAI & Colon Length & Spleen Weight contains the clinical data of UC mice. The JPG files WB1 and WB2 are representative WB gel bands existing in this article. [file 6257778.f1.zip › 6257778.f1/Figures-other 5 alkaloids molecular docking.pdf]
