# Supplementary figures and images for: Alkaloids from Aconitum carmichaelii Alleviates DSS-Induced Ulcerative Colitis in Mice via MAPK/NF-κB/STAT3 Signaling Inhibition
Source: Evid Based Complement Alternat Med. 2022 May 31;2022:6257778. doi: 10.1155/2022/6257778 (PMC9173982; doi:10.1155/2022/6257778)

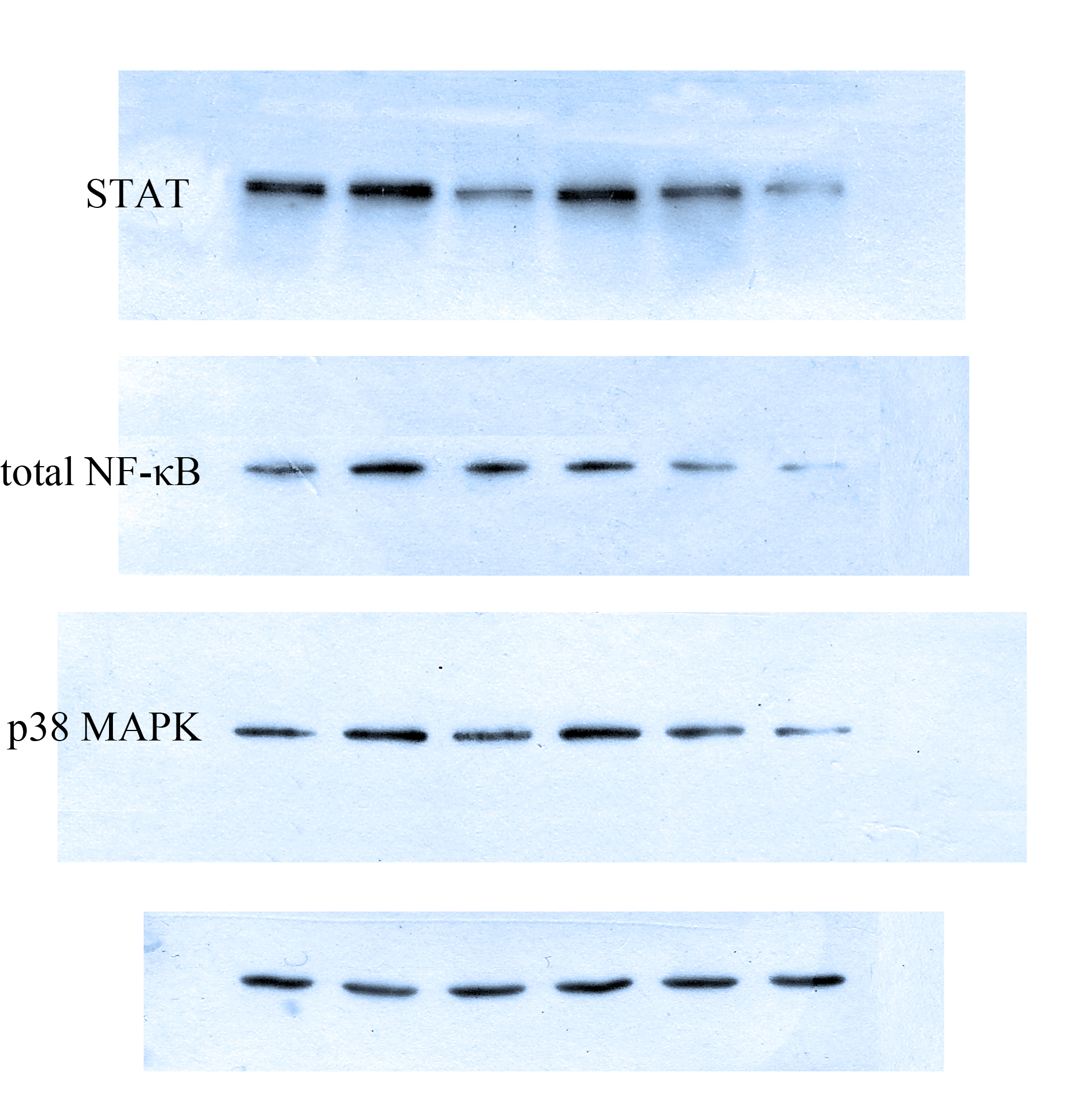

Supplement: Supplementary Materials — The PDF file Figures-other 5 alkaloids molecular docking contains molecular docking simulations among aconitine, hypaconitine, mesaconitine, benzoylaconine, benzoylmesaconine, and MAPK/NF-κB/STAT3 proteins. The PDF file Change of body weight & DAI & Colon Length & Spleen Weight contains the clinical data of UC mice. The JPG files WB1 and WB2 are representative WB gel bands existing in this article. [file 6257778.f1.zip › 6257778.f1/WB1-jpg.jpg]

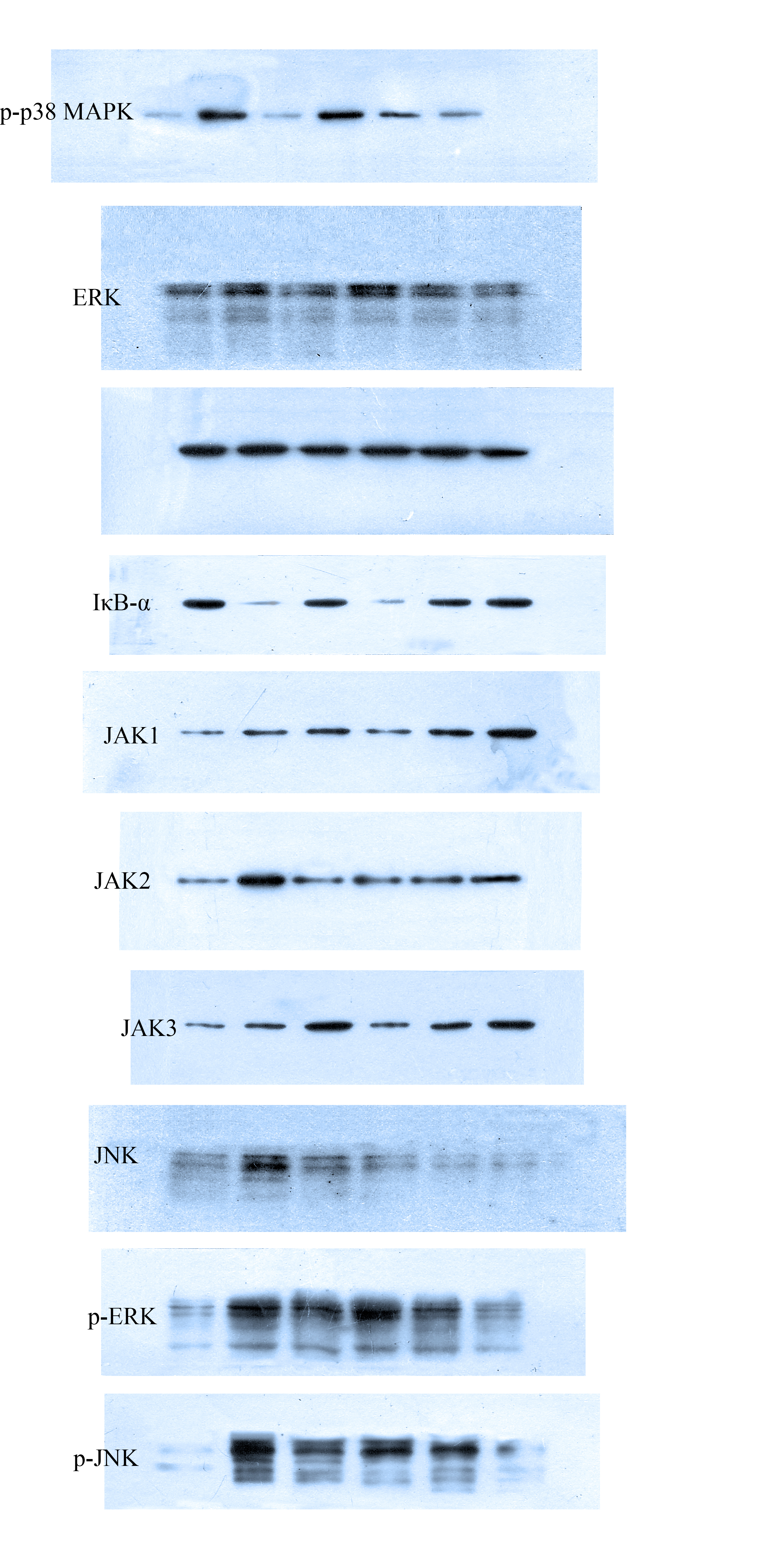

Supplement: Supplementary Materials — The PDF file Figures-other 5 alkaloids molecular docking contains molecular docking simulations among aconitine, hypaconitine, mesaconitine, benzoylaconine, benzoylmesaconine, and MAPK/NF-κB/STAT3 proteins. The PDF file Change of body weight & DAI & Colon Length & Spleen Weight contains the clinical data of UC mice. The JPG files WB1 and WB2 are representative WB gel bands existing in this article. [file 6257778.f1.zip › 6257778.f1/WB2-jpg.jpg]
